# *Lasia spinosa* Chemical Composition and Therapeutic Potential: A Literature-Based Review

**DOI:** 10.1155/2021/1602437

**Published:** 2021-12-28

**Authors:** Rajib Hossain, Cristina Quispe, Jesús Herrera-Bravo, Md. Shahazul Islam, Chandan Sarkar, Muhammad Torequl Islam, Miquel Martorell, Natália Cruz-Martins, Ahmed Al-Harrasi, Ahmed Al-Rawahi, Javad Sharifi-Rad, Manshuk Ibrayeva, Sevgi Durna Daştan, Mohammed M. Alshehri, Daniela Calina, William C. Cho

**Affiliations:** ^1^Department of Pharmacy, Life Science Faculty, Bangabandhu Sheikh Mujibur Rahman Science and Technology University, Gopalganj 8100, Bangladesh; ^2^Facultad de Ciencias de la Salud, Universidad Arturo Prat, Avda. Arturo Prat 2120, Iquique 1110939, Chile; ^3^Departamento de Ciencias Básicas, Facultad de Ciencias, Universidad Santo Tomas, Chile; ^4^Center of Molecular Biology and Pharmacogenetics, Scientific and Technological Bioresource Nucleus, Universidad de La Frontera, Temuco 4811230, Chile; ^5^Department of Nutrition and Dietetics, Faculty of Pharmacy, And Centre for Healthy Living, University of Concepción, 4070386 Concepción, Chile; ^6^Universidad de Concepción, Unidad de Desarrollo Tecnológico, UDT, Concepción 4070386, Chile; ^7^Department of Biomedicine, Faculty of Medicine, University of Porto, Alameda Prof. Hernâni Monteiro, Porto, Portugal; ^8^Institute for Research and Innovation in Health (i3S), University of Porto, Porto, Portugal; ^9^Institute of Research and Advanced Training in Health Sciences and Technologies (CESPU), Rua Central de Gandra, 1317, 4585-116 Gandra PRD, Portugal; ^10^TOXRUN – Toxicology Research Unit, University Institute of Health Sciences, CESPU, CRL, 4585-116 Gandra, Portugal; ^11^Natural and Medical Sciences Research Centre, University of Nizwa, Birkat Almouz, 616, Oman; ^12^Facultad de Medicina, Universidad del Azuay, Cuenca, Ecuador; ^13^Faculty of Science and Technology, The Caspian University of Technology and Engineering Named after Sh. Yessenov, Aktau, Kazakhstan; ^14^Department of Biology, Faculty of Science, Sivas Cumhuriyet University, 58140 Sivas, Turkey; ^15^Beekeeping Development Application and Research Center, Sivas Cumhuriyet University, 58140 Sivas, Turkey; ^16^Pharmaceutical Care Department, Ministry of National Guard-Health Affairs, Riyadh, Saudi Arabia; ^17^Department of Clinical Pharmacy, University of Medicine and Pharmacy of Craiova, 200349 Craiova, Romania; ^18^Department of Clinical Oncology, Queen Elizabeth Hospital, Kowloon, Hong Kong

## Abstract

*Lasia spinosa* (L.) is used ethnobotanically for the treatment of various diseases, including rheumatoid arthritis, inflammation of the lungs, bleeding cough, hemorrhoids, intestinal diseases, stomach pain, and uterine cancer. This review is aimed at summarizing phytochemistry and pharmacological data with their molecular mechanisms of action. A search was performed in databases such as PubMed, Science Direct, and Google Scholar using the keywords: “*Lasia spinosa*,” then combined with “ethnopharmacological use,” “phytochemistry,” and “pharmacological activity.” This updated review included studies with in vitro, ex vivo, and in vivo experiments with compounds of known concentration and highlighted pharmacological mechanisms. The research results showed that *L. spinosa* contains many important nutritional and phytochemical components such as alkanes, aldehydes, alkaloids, carotenoids, flavonoids, fatty acids, ketones, lignans, phenolics, terpenoids, steroids, and volatile oil with excellent bioactivity. The importance of this review lies in the fact that scientific pharmacological evidence supports the fact that the plant has antioxidant, anti-inflammatory, antimicrobial, cytotoxic, antidiarrheal, antihelminthic, antidiabetic, antihyperlipidemic, and antinociceptive effects, while protecting the gastrointestinal system and reproductive. Regarding future toxicological and safety data, more research is needed, including studies on human subjects. In light of these data, *L. spinosa* can be considered a medicinal plant with effective bioactives for the adjuvant treatment of various diseases in humans.

## 1. Introduction

Traditional, herbal, and Ayurvedic medicine comprise an important and prestigious form of treatment for various diseases and conditions in different locations all over the world from the beginning of human civilization on Earth [1]. Several plants and their corresponding preparations have been used for various therapeutic purposes for a long time ago. The history of traditional, herbal, and Ayurvedic medicine is the eldest to establish a treatment pattern [1].

*Lasia spinosa* (L.) Thwaites, often known as Chengmora/Sibru in Assames, Kata-kachu in Bengali, Janum-Saru in Manipuri, Kohila/Mahakohila/Engilikohila in Sri Lanka, Zawangzang in Mizoram, and Laksmana in Sanskrit [2–4], belongs to the *Araceae* family [5]. It is found in Asia-Bangladesh, China, the Indian subcontinent, Myanmar, Thailand, Indo-China, Indonesia, and Papua New Guinea [6].

Briefly, *L. spinosa* is an aquatic or terrestrial plant, short-stemmed spiny heirs with underground rhizome that usually occurs in wet forests, open marshes, wetlands, or in permanently standing water [7]. *L. spinosa* is a large marsh plant with the stem stout 1 m high and the leaves broadly arrow-shaped in outlines, 20-30 cm long deeply divided into 4-6 pairs of narrow side lobes. The petiole is 30-40 cm long, veins beneath the petiole and peduncle prickly [8]. Morpho-anatomical feature of *L. spinosa* has been recently reported by Lakshmi et al. [9]. The plant is harvested from the wild for its edible leaves and various medicinal uses. Sometimes, it is also cultivated as a vegetable crop along ponds margins [10]. Recently, it has been reported that *Fusarium fujikuroi* caused leaf spot on *L. spinosa* in China [11].

With regards to their biological effects, the tender leaves and rhizomes of this plant, used as a vegetable and in indigenous medicine, have been recommended for a variety of conditions [12–15].

Given the multiple potentialities of this plant, this review provides up-to-date data on *L. spinosa* chemical composition and biological effects based on the scientific reports found in the databases.

## 2. Review Methodology

In this study, the literature on botanical classification of *L. spinosa*, ethnomedicinal applications, secondary metabolites, biological properties were compiled, reviewed, and summarized. For the compilation of all written papers on this species, scientific search engines such as PubMed, ScienceDirect, SpringerLink, Web of Science, Scopus, Wiley Online, Scifnder, and Google Scholar have been used. In this study, the literature on botanical classification of Lasia spinosa, ethnomedicinal applications, secondary metabolites, and biological properties were compiled, reviewed, and summarized. For searching, the next MeSH terms were used: “Phytotherapy”, “Plants”, “Medicinal”, “Plant Extracts/administration & dosage”, “Plant Extracts/isolation & purification”, “Plant/chemistry”, “Structure-Activity Relationship”, “Disease Models”, “Animal”, and “Plant Extracts/toxicity”. Using the Chemsketch version 12.01 program, chemical structures were drawn. The scientific names of the plants have been verified according to the PlantList [16, 17].

Inclusion criteria: works published in English on Lasia spinosa that highlighted the following data: chemical compounds isolated from each genus, preclinical pharmacological research highlighting molecular mechanisms, and *in vitro/in vivo* pharmacological studies that contained the concentration and dose at which the chemical compounds studied were pharmacologically active and toxicological data. The most important results obtained were summarized in the tables.

Exclusion criteria: abstracts, letters to the editor, papers in languages other than English, studies that did not have dose-effect correlations, and studies that did not have proven molecular mechanisms which underline the pharmacology.

## 3. Ethnopharmacology

*L. spinosa* is a medicinally important plant, traditionally used by different ethnic communities all over the world. There are various reports on *L. spinosa* medicinal and economical properties. Often used for treating colic, tuberculosis of lymph nodes, swollen lymph nodes, rheumatism/rheumatoid arthritis, injuries, snake bites, and insect bites, this plant is also recommended as effective for the treatment of sore throat, constipation, to purify the blood, on lung inflammation, bleeding cough, and uterine cancer [14, 18–20]. Rhizomes (roots) are most often used as a remedy for hemorrhoids in Sri Lanka and Malays and to confer protection for some of the above conditions, because of their high fibre content and antioxidant compounds [15].

Besides, leaves and stalks have demonstrated profound antihelminthic, anticestode, and antinematode efficacy [12, 15, 18, 19, 21, 22]. The root decoction is also useful in gastrointestinal diseases and stomachache [4], while also stimulating liver function [22]. Paste from tender leaves is externally used in burns [4].

## 4. Phytochemical Profile

*L. spinosa* whole plant contains several essential phytochemicals, including alkaloids, flavonoids, tannin, saponins, steroids, terpenoids, and varying amounts of micronutrients, like zinc (Zn), magnesium (Mg), calcium (Ca), iron (Fe), copper (Cu), manganese (Mn), and molybdenum (Mo) (Table 1).

Nutritional analysis of *L. spinosa* showed that it contains proteins (17.6 kcal/100 g), fats (1.16 kcal/100 g), and carbohydrates (35.7 kcal/100 g), with a nutritive value of 224 kcal/100 g [2, 23]. In another study, the protein, fats, and carbohydrate content on a dry weight basis were 17.9, 3.8, and 45.5 g/100 g edible portion for protein, fats, and carbohydrate, respectively, for *L. spinosa* leaf, with a nutritive value of 288.5 kcal/100 g [24]. *L. spinosa* roots/rhizome contains dietary fibre, Ca, and provitamin A carotenoids [18, 25]. *L. spinosa* leaf contains 15.4 g of fibre, 250 mg of Ca, 19.2 mg of Fe, and 455 mg of vitamin C for 100 g edible portion on a dry weight basis [24, 26].

In fresh weight, other studies reported content of proteins, fats, and carbohydrates of 3.68 ± 0.28, 0.44 ± 0.03, and 4.78 ± 0.38 g/100 g, respectively, the mineral content of 158.08 ± 3.98, 321.73 ± 7.00, 73.17 ± 2.37, 53.86 ± 3.86, and 0.92 ± 0.08 mg/100 g for Ca, K, P, Mg, and Fe, and 2.99 ± 0.11 and 0.28 ± 0.01 mg/100 g of vitamins C and E, respectively [27].

When specifically addressing the different extracts prepared from *L. spinosa*, hexane extracts leaves and root contains the alkaloid berberine [28], lignan (e.g., lyoniresinol, meridional, secoisolariciresinol; 5,5′-dimethoxysecoiso-lariciresinol; 2-(4-hydroxy-3,5-dimethoxybenzyl)-3-(4-hydroxy-3-methoxybenzyl)-1,2-butanediol; (7′S,8S,8R)-4,4′-dihydroxy-3,3′5,5′-tetra methoxy-7′,9-eproxylignan-9′-ol-7-one; 5,5′-dimethoxy-lariciresinol; 5′-methyoxlariciresinol, dihydrodehydrodiconifery alcohol; syringaresinol) [27–29], aldehyde (e.g., p-hydroxy benzaldehyde) [30], phenolic (e.g., procyanidin A1) [31] and other compounds (e.g., 4-hydroxybenzoic acid, 2-(4′- methoxyphenyl)-ethanol, 4-methoxyphenyl alcohol, 1-tetracosane) [30], from stem carotenoids (e.g., *α*-carotene, *β*-carotene, *β*-carotene-5,6, 5′, 6′-diepoxide; 5, 6, 5′, 6′-diepoxy-5, 8, 5′,8′-tetrahydro-*β*, cis-neoxanthin, and unidentified carotenoids I, II, III, and IV) are isolated [24, 32, 33].

The aerial parts of *L. spinosa* contain terpenoids (e.g., limonene, aqualene, caryophyllene), volatile oil (e.g., methyl octadec-6,9-dien-12-ynoate, *α*-glyceryl-linolenate *α*-pinene, *α*-selinene, camphene, *δ*-3-carene, camphor) [21, 34], phenolic compounds (e.g., 4-hydroxybenzoic acid, morin, cinnamic acid, syringic acid, gentisic aid) [21, 28, 34], fatty acids (e.g., methyl ester of oleic acid, palmitic acid, stearic acid, epoxyoleic acid) [34], steroids (e.g., spinasterone, *β*-sitosterol, *γ*-sitosterol, stigmasterol, campesterol, crinosterol) [21, 34], alkane (e.g., hexatriacontane and heptacosane) [35].

The whole plant contains phenolics (e.g., gentisic acid, isovanilic acid, syringic acid, chlorogenic acid, p-hydroxy benzoic acid, (+)-catechin) [28], flavonoids including flavonoid glycosides and flavonoid aglycones (e.g., vitexin, vitexin-2”-O-*β*-D-glucopyranoside; isorhamnetin 3-O-rutinoside, morin, apigenin, 3′-methyl-quercetin-3-O-*α*-L-rhamnopyranosyl-(1/6) *β*-D-glucopyranoside; triglochinin) [28, 32, 35], and ketone (e.g., hexahydrofarnesyl acetone) (21).

**Figure 1:**
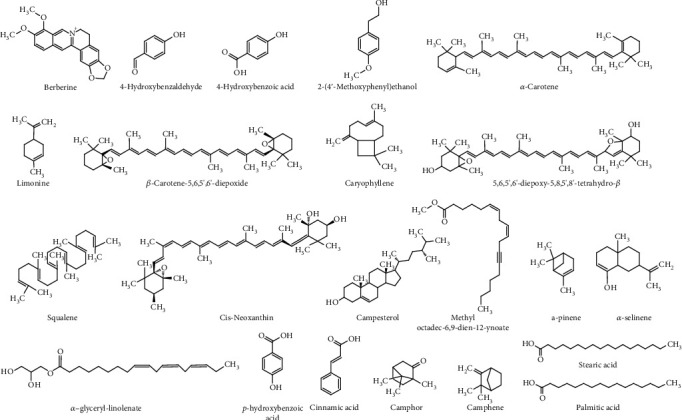
Chemical structures of the most important phytochemicals found in different parts of *Lasia spinosa.*

The chemical structures of such compounds are shown in Table 2 and Figure 1.

## 5. Pharmacological Properties: Mechanisms and Targeted Molecular Pathways

### 5.1. Antioxidant

Oxidative stress is the basis of premature ageing of the body, the basis of disease, and is triggered by free radicals [29] more precisely occurs as a result of the imbalance between the amount of reactive oxygen produced in the body and its ability to eliminate it [30, 31]. Oxidative stress can be alleviated by approaching a balanced lifestyle that includes a healthy diet and sports [32]. Physical exercise reduces cellular oxidation by deep oxygenating tissues, eliminating stress, and relaxing the body [33]. On the other hand, the diet has a very important role, and the best treatment against oxidative stress is antioxidants [34]. They are found in many herbs and can kill free radicals [35, 37]. Medicinal plants usually contain a high level of antioxidants that can counteract the oxidative stress process linked to a disease [38, 39].

The free radical scavenging activity of *L. spinosa* leaves extracts on 1,1-diphenyl-2-picrylhydrazyl (DPPH) had been assessed and showed significant antioxidant activities [40]. The ethyl acetate fraction showed the highest free radical scavenging activity (IC_50_ = 16.42 *μ*g/mL) when compared to the positive control-butylated hydroxytoluene (BHT). At the same time, the aqueous fraction also exhibited moderate antioxidant potential (IC_50_ = 73.20 *μ*g/mL) [40]. In DPPH and ABTS assay, ethanol extract (leaves) showed antioxidant activity (SC_50_ = 17.25 *μ*g/mL and 16.47 *μ*g/mL, respectively). Antioxidant activity is due to the presence of high levels of polyphenolic compounds [38]. In a study performed with different extracts of *L. spinosa* aerial parts, the highest free radical scavenging activity (DPPH) was stated to the methanol extract (IC_50_ = 0.48 ± 0.04 *μ*g/mL), whereas in the metal chelating activity of ferrous ions (Fe^2+^) assay, the highest activity was observed for hexane extract (IC_50_ = 0.55 ± 0.08 *μ*g/mL) [23]. In another study, the antiradical activity (1/EC_50_) of *L. spinosa* leaf determined by the DPPH method was 0.1 [24]. The same study reported a total phenolic content of 6.4 mg gallic equivalents/g and total flavonoid content of 4.4 retinol equivalent in *L. spinosa* leaf [24]. In other studies, *L. spinosa* showed a total phenolic content of 2.1 mg gallic equivalents/g and low antioxidant activity, through ferric reducing antioxidant power (FRAP) and oxygen radical absorbance capacity (ORAC) assays, in comparison to indigenous vegetables from Southern Thailand, such as young cashew leaves (*Anacardium occidentale* L.) and Mon-pu (*Glochidion zeylanicum* (Gaertn.) A.Juss.) [27].

### 5.2. Anti-Inflammatory

Inflammation is part of the complex biological response of body tissues to harmful stimuli such as pathogens, damaged cells, or irritants [41] and a protective response involving immune system cells, molecular mediators, among others [42–44].

In lipopolysaccharide-induced RAW 264.7 macrophages, the anti-inflammatory activity of *L. spinosa* leaf extract was addressed [45], is stated that it can activate the nuclear factor- (NF-) *κ*appa B, and nuclear factor erythroid 2-related factor 2/heme-oxygenase-1 (Nrf2/HO-1) pathways and to suppress mitogen-activated protein kinase (MAPK) and phosphoinositide-3-kinase/protein kinase B (PI3K/Akt) pathways. Furthermore, *L. spinosa* leaf extract suppresses the upregulation enzyme iNOS (NOS2), COX2, and proinflammatory cytokines (TNF-*α*, IL-1*β*, and IL-6) and increases cytokines (IL-10) which produced anti-inflammatory effect [40].

**Figure 2:**
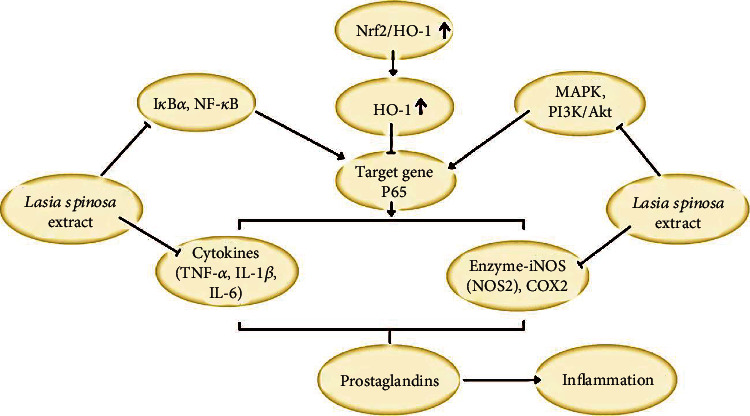
Diagram with molecular mechanisms of anti-inflammatory effect of *Lasia spinosa.*

In another study, the anti-inflammatory activity of *L. spinosa* hydroalcoholic extract in xylene-induced ear oedema model mice was assessed, being stated a significant inhibitory effect on oedema formation 17.1% at 250 mg/kg and 27.9% at 500 mg/kg. An inhibitory potential was also stated in a carrageenan-induced paw oedema model rat, and it was highest at 3 h, with 26.72% inhibition at 250 mg/kg and 38.70% at 500 mg/kg, when compared to the standard drugs (diclofenac sodium (10 mg/kg): 29.52%, and phenylbutazone (100 mg/kg): 40.47%) (Figure 2) [46].

### 5.3. Antimicrobial

An antimicrobial agent is that able to kill or stop microorganisms' growth. For that, antibacterial and antifungals are used to fight bacterial and fungal infections, respectively [47–49]. Specifically addressing antibacterial, their prolonged use is closely related to a marked decrease in the number of enteric bacteria, thus, having a major negative impact on health and wellbeing [50, 51]. In this sense, the consumption of probiotics and a prebiotics-rich diet may help to replace the destroyed gut microbiota [52]. Stool transplants may also be proposed for patients with difficulty in recovering from prolonged antibiotic treatment, as for recurrent *Clostridium difficile* infections [53, 54].

The organic extracts (hexane, chloroform, ethyl acetate; 300 *μ*g/disc) and essential oil of *L. spinosa* aerial parts showed potent antibacterial activity against *Escherichia coli*, methicillin-resistant *Staphylococcus aureus*, *Klebsiella pneumoniae*, *Pseudomonas aeruginosa*, and *Enterococcus faecalis* in comparison of standard antibiotics (tetracycline 30 *μ*g/disc, streptomycin 30 *μ*g/disc, and erythromycin 15 *μ*g/disc), except for methanol extract [23]. In another study, *L. spinosa* leaves methanol extract showed moderate antimicrobial properties against *Bacillus subtilis*, *E. coli*, *Bacillus cereus*, *S. aureus*, *Candida albicans*, *Aspergillus niger*, and *Vibrio para hemolyticus* at 400 *μ*g/disc by disc diffusion assay [36]. In other studies, the methanol extract of *L. spinosa* edible parts did not show antimicrobial activity against *C. albicans* [46].

### 5.4. Cytotoxic

Cytotoxicity destroys cancer cells or prevents them from multiplying [55–57]. This cytotoxicity is done in different ways: some bioactive compounds can affect the genetic material of cells, and others act by blocking the access of malignant cells to the nutrients needed for division and multiplication [58, 59].

The cytotoxic potential of *L. spinosa* extracts has also been assessed. Brine shrimp lethality bioassay technique was applied to determine the cytotoxic potential of crude extracts. The aqueous extract from *L. spinosa* leaves showed moderate cytotoxicity (LC_50_ = 98.66 *μ*g/mL) in brine shrimp lethality bioassay [40], while the methanol extract from the whole plant led to significant cytotoxic effects (IC_50_ = 13.49 *μ*g/mL) on brine shrimp [36].

### 5.5. Antidiarrheal

In the castor oil-induced diarrheal mice model, both standard antimotility drug loperamide and hydroalcoholic extract from *L. spinosa* root significantly reduced the number of stools in a dose-dependent manner compared to the negative control group. The mean number of stools found was 11.6 for 250 mg/kg and 8.2 for 500 mg/kg b.w., whereas for the standard drug (5 mg/kg) was of 5.6.*L. spinosa* root extract at 250 mg/kg b.w, causing a little increase in the latent period, but at 500 mg/kg b.w. led to a significant increase [46]. *L. spinosa* root extract exhibited a potent antidiarrheal activity, supporting their traditional use for diarrhea.

### 5.6. Antihelminthic

Helminthic infections continue to be the major people's health hazard, especially in those living in tropical developing countries [60]. *L. spinosa* leaves methanol extract significantly exhibited paralysis and triggered worms' death, especially at high doses (100 mg/mL) against *Pheritimaposthuma* [61], in a hymenolepisdiminuta–rat animal model [12] and infected mice with *Trichinellaspiralis* (800 mg/kg; *p.o.*) [19].

### 5.7. Antidiabetic

Diabetes mellitus is a chronic metabolic disease with numerous complications, like retinopathy, neuropathy, and peripheral vascular insufficiency. Several synthetic agents are available for diabetes treatment, but several side effects have been reported [62]. Plant-based medicinal products have been used since ancient times to manage diabetes in traditional medicine in many countries all over the world. *L. spinosa* stem hydroalcoholic extract have revealed antidiabetic activity at 200 and 400 mg/kg (p.o.) in dexamethasone (10 mg/kg s.c.)-induced diabetic albino rats by preventing serum glucose levels rise triggered by dexamethasone [63] and significantly reducing the triglycerides levels [64]. This extract also ameliorated hyperglycemia, and it likely has greater therapeutic potential as they may also exert beneficial effects on the clinical course of noninsulin-dependent diabetes mellitus (NIDDM), hypertension, and coronary artery disease [63]. A study developed by Shafie et al. [65] showed inhibitory effects of different parts (leaves, stems, and roots) of *L. spinosa* extracts (aqueous hot/cold, ethanol) against pancreatic lipase, *α*-amylase, and *α*-glucosidase.

### 5.8. Antihyperlipidemic

*L. spinosa* leaves have also the potential to prevent hyperlipidemia-induced pancreatitis in rats at concentrations of 400 and 800 mg/kg (p.o.) while exerting cardioprotective effects by significantly increasing serum high-density lipoprotein-cholesterol (HDL-c) at 100 mg/kg, p.o., and Triton-X 100 at 480 mg/kg, i.p. in an induced hyperlipidemic animal model [3].

### 5.9. Antinociceptive

Antinociception is the action or process of blocking the detection of a painful or injurious stimulus by sensory neurons, and antinociceptives are agents that block painful stimulus [66, 67]. The acetic acid-induced writhing test is used for detecting both central and peripheral analgesia, whereas the hot plate is most sensitive to centrally acting analgesics [68]. In acetic acid-induced writhing and hot plate-induced pain in mice the hydroalcoholic extract of *L. spinosa* roots revealed antinociceptive activity in mice, being stated 37% and 50% writhing inhibition at 250 mg/kg and 500 mg/kg b.w., respectively, while increased pain threshold [46]. On the other hand, the methanol extract from *L. spinosa* leaves at 400 mg/kg led to a significant decrease in the number of writhes and elongated the reaction time in the acetic acid writhing method and radiant heat tail flicking method, respectively [69].

### 5.10. Gastroprotective

A study revealed that *L. spinosa* leaves ethanol extract has gastroprotective effects. In albino rats with indomethacin (5 mg/kg, p.o.) and cold restrain stress-induced ulcers, 3 doses (100, 200, and 400 mg/kg, p.o.) of *L. spinosa* extract were tested, with gastroprotective effects being mainly conferred by the extractability to create a defensive layer in stomach, through scavenging free radicals and inhibiting lipid peroxidation [70]. In gastric secretion studies, *L. spinosa* significantly evidenced a tendency to decrease gastric juice, free acidity, and total acidity [70]. Thus, after isolation of the individual compounds present in the extract, those responsible for the observed effect can be used both to treat ulcers and to reduce their severity.

### 5.11. Effect on Reproductive Activity

Testosterone plays an important role in Sertoli and Leydig cell proliferation and hyperplasia that can increase the testis size [71]. Testosterone is also involved in spermatogenesis and the growth and development of testis and male accessory reproductive glands [64]. The hydroalcoholic extract of *L. spinosa* rhizomes was revealed to be able to increase the serum testosterone levels and sperm count in male rats at 40 g/kg b.w. These data were confirmed afterward by an increase in testicular weight and sperm count, with the increase in the absolute weight of testis being attributed to the elevation of androgen biosynthesis leading to an increase in serum testosterone levels [64].

**Figure 3:**
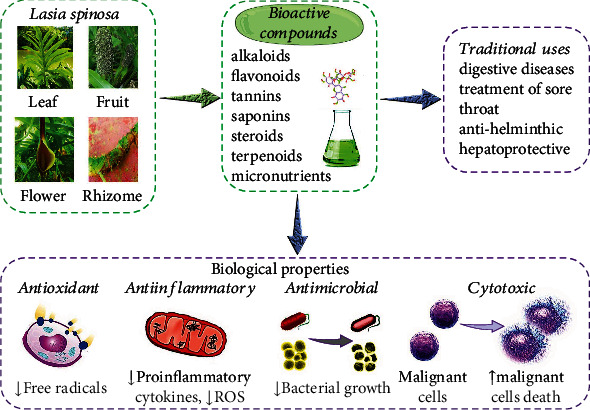
Summarized scheme with traditional uses and the most representative biological properties of *L. spinosa.*

The most important pharmacological properties are summarized in Table 3 and Figure 3.

## 6. Toxicological Data and Clinical Gaps

Oral administration in an acute analysis of 5, 10, 20, and 40 gm/kg of an extract, there were no mortality or physiological changes demonstrated. In the subchronic assay for 28 gm/kg, administration of 5 or 20 gm/kg of extract for 28 gm/kg, no animal deaths were announced that day. No differences in haematological parameters were noticed in either case [52].

Therapeutic limitation of natural bioactive compounds from *Lasia spinosa* results from the relatively reduced bioavailability of bioactive compounds. In addition, numerous interactions with other prescription drugs may occur. Interactions between medicinal plants interfere with the metabolism or elimination of the drug/chemotherapy from the body. Drug metabolism/elimination is mediated by enzymes that metabolize drugs in the cytochrome P450 (CYP) family and drug transport proteins. These interactions can change the concentration of drugs in the body [72].

Interactions between plants and drugs may occur due to inhibition or activation by plant phytochemicals of CYP enzymes or drug transport proteins that metabolize the drug [73]. Some therapeutic pharmacological agents must be activated by CYP to be effective. Once CYPs are inhibited, such drugs that need to be activated will be ineffective. There may be interactions between plants and drugs that lead to increased elimination of drugs due to CYP activation, which could lead to subtherapeutic exposure to drugs and could lead to therapy failure [74]. Some plant-drug interactions due to CYP inhibition may lead to the accumulation of cytotoxic drugs due to delayed clearance and may increase drug toxicity due to high doses of drugs. Cancer patients are already taking several medications at the same time due to other conditions associated with cancer and comorbidities, which present a risk of drug interactions [75]. The use of herbs/herbal products may further increase the risk of these potentially harmful interactions that interfere with the impact of the drug.

## 7. Overall Conclusions and Future Perspectives

Natural plant sources have contributed to many drug developments. In this study, we comply with the traditional uses, pharmacological properties, and chemical constituents of *L. spinosa*, information that can be useful for further research. Many phytochemicals present in *L. spinosa* may be responsible for its biological effects in various test systems, but more studies are needed to identify and characterize the active compounds responsible for the pharmacological activities of this hopeful medicinal plant. Future directions must be oriented to toxicological studies which are scarce, and there are necessary new reports to ensure the safety of this plant. Besides, clinical studies are required to confirm the preclinical biological effects in humans.

## Figures and Tables

**Table 1:** Amounts of micronutrients of *Lasia spinosa* in ppm (parts per million) [2].

Elements	Amounts (ppm)
Zn	7.442 ± 0.01
Mg	6.228 ± 0.11
Fe	17.06 ± 0.87
Cu	0.316 ± 0.02
Mn	1.334 ± 0.08
Mo	1.180 ± 0.06

**Table 2:** Phytochemical profile of *Lasia spinosa.*

Plant parts	Phytochemical class	Compounds	Ref.
Leave	Alkaloids	Berberine	[25]

Leaf and root/rhizome	Aldehyde	*p*-Hydroxy benzaldehyde	[26]
	Other compounds	4-Hydroxybenzoic acid, 2-(4′-methoxyphenyl)-ethanol, 4-methoxyphenethyl alcohol, 1-tetracosane	

Stem	Carotenoid	*α*-Carotene, *β*-carotene, *β*-carotene-5,6, 5′, 6′-diepoxide; 5, 6, 5′, 6′-diepoxy-5, 8, 5′,8′-tetrahydro-*β*, cis-neoxanthin	[24, 31] Priyadarshani and Jansz, [25]

Aerial parts	Terpinoid	Limonene, *β*-elemene, squalene, caryophyllene	[21]; Rahman et al., [23]
	Volatile oil	Methyl octadec-6,9-dien-12-ynoate, *α*-glyceryl-linolenate *α*-pinene, *α*-selinene, camphene, *δ*-3-carene, camphor	[21]; Rahman et al., [23]
	Phenolics	4-Hydroxybenzoic acid, morin, cinnamic acid, syringic acid, gentisic acid	Rahman et al., [23]; [21, 28]
	Fatty acids	Methyl ester of oleic acid, palmitic acid, stearic acid, epoxyoleic acid	Rahman et al., [23]
	Steroid	Spinasterone, *β*-sitosterol, *γ*-sitosterol, stigmasterol, campesterol, crinosterol,	Rahman et al., [23]; [21]
	Alkane	Hexatriacontane, heptacosane	Rahman et al., [23]

Root/rhizome	Lignan	Lyoniresinol, meridinol, secoisolariciresinol; 5,5′-dimethoxysecoiso-lariciresinol; 2-(4-hydroxy-3,5-dimethoxybenzyl)-3-(4-hydroxy-3-methoxybenzyl)-1,2-butanediol; (7′S,8S,8R)-4,4′-dihydroxy-3,3′5,5′-tetramethoxy-7′,9-eproxylignan-9′-ol-7-one; 5,5′-dimethoxy-lariciresinol; 5′-methyoxlariciresinol, dihydrodehydrodiconifery alcohol; syringaresinol	Alam et al., [36]; [28, 29, 32]
	Phenolic	Procyanidin A1	[30]

Whole plant	Phenolic	Gentisic acid, isovanilic acid, syringic acid, chlorogenic acid, p-hydroxy benzoic acid, (+)-catechin	[28]
	Flavonoids (glycosides and aglycones)	Vitexin, vitexin 2”-O-*β*-D-glucopyranoside; isorhamnetin 3-O-rutinoside, morin, apigenin, 3′-methyl quercetin-3-O-*α*-L-rhamnopyranosyl-(1/6) *β*-D-glucopyranoside; triglochinin	[27, 28, 31]
	Ketone	Hexahydrofarnesyl acetone	[21]

**Table 3:** Pharmacological activities of *Lasia spinosa*.

Activity	Sources	Test system	Dose tested	Positive value	Results	Ref.
Antioxidant	Ethyl acetate extract (leaves)	DPPH	IC_50_ = 73.20 *μ*g/mL	BHT IC_50_ = 23.19 *μ*g/mL	Moderate potential	[40]
	Ethanol extract (leaves)	DPPH ABTS	DPPH, IC_50_ = 17, 25 *μ*g/mL; ABTS SC_50_ = 16.47 *μ*g/mL	Vitamin C Dose = 5.38 *μ*g/mL Trolox Dose = 3.17 *μ*g/mL	↑ antioxidant activity due to the presence of high levels of polyphenolic compounds	[45]
	Hexane, chloroform, ethyl acetate, and methanol extracts (aerial parts)	DPPH Chelating activity of ferrous ions (Fe^2+^)	IC_50_ = 0.48 ± 0.04 *μ*g/mL (methanolic) IC_50_ = 0.55 ± 0.08 *μ*g/mL (hexane)	Not studied	↑ antioxidant activity	[23]

Anti-inflammatory	Ethanol extract (leaves)	Lipopolysaccharide-induced RAW 264.7 macrophages *in vitro*	Dose = 50, 100, 200, 400 *μ*g/mL	L-NAME Dose = 100 *μ*M	↑ NF-*κ*B, ↑ Nrf2/HO-1 ↓MAPK, ↓ PI3K/Akt	[45]
	Hydroalcoholic extract (roots)	Carrageenan-induced paw edema model rats and xylene-induced ear edema mice *in vivo*	Dose = 250, 500 mg/kg bw, i.p. *n* = 6	Nalbuphine Dose = 10 mg/kg	↓oedema formation	[46]

Antimicrobial	Methanol extract (leaves)	Disc diffusion assay *in vitro*	Dose = 400 *μ*g/disc	Kanamycin Dose = 30 *μ*g/disc	Moderate antimicrobial activity against *Bacillus subtilis*, *Escherichia coli*, *Bacillus cereus*, *Staphylococcus aureus*, *Candida albicans*, *Aspergillus niger*, and *Vibrio parahemolyticus*	[36]
	Hexane, chloroform, ethyl acetate, and methanol extracts (aerial parts) and essential oil	Disc diffusion assay *in vitro*	Dose = 300 *μ*g/disc	Tetracycline Dose = 30 *μ*g/disc Streptomycin Dose = 30 *μ*g/disc Erythromycine Dose = 15 *μ*g/disc	Potent antibacterial activity against *Escherichia coli*, methicillin-resistant *Staphylococcus aureus*, *Klebsiella pneumoniae*, *Pseudomonas aeruginosa*, and *Enterococcus faecalis*, with the exception of methanolic extract	[23]

Cytotoxicity	Hydromethanolic extract (leaves)	Brine shrimp lethality bioassay *in vitro*	LC_50_ = 98.663 *μ*g/mL	Vincristine sulphate LC_50_ = 0.544 *μ*g/ml	Moderate cytotoxic effect	[40]
	Methanol extract (whole plant)	Triton-X 100 (480 mg/kg, i.p.) induced hyperlipidemic rat model *in vivo*	IC_50_ = 13.49 *μ*g/mL	Vincristine sulphate LC_50=_20 *μ*g/ml	↑cytotoxicity	[36]
			Dose = 200, 400, 800 mg/kg, p.o. *n* = 6	Triton-X 100 Dose = 480 mg/kg	↓triglycerides, ↓LDL-C ↓VLDL-C	

Antidiarrheal	Hydroalcoholic extract (roots)	Castor oil-induced diarrhea mice model *in vivo*	250 and 500 mg/kg b.w., i.p. *n* = 6	Loperamide Dose = 5 mg/kg	↓number of stools ↑latent period of diarrhea	[46]

Anthelmintic	Methanol extract (leaves)	*Pheritima posthuman in vivo*	Dose = 25, 50, 100 mg/mL, *n* = 6	Albendazole Dose = 10 mg/ml	↑paralysis, ↑worm death, especially at 100 mg/ml	[61]
	Extract (leaves)	*Hymenolepis diminuta* rat model *in vivo*	Dose = 200, 400, 800, 1600 mg/kg, p.o., *n* = 6	Praziquantel Dose = 5 − 10 mg/kg	↓eggs per gram of feces ↓worm recovery rates	[12]

Antidiabetic	Hydroalcoholic extract (stem)	Dexamethasone 10 mg/kg s.c. induced diabetes rats *in vivo*	Dose = 200, 400 mg/kg, p.o. *n* = 6	Dexamethasone dose = 10 mg/kg	↑antidiabetic activity	[63]

Antihyperlipidemic	Methanol extract (leaves)	Cholestero,l 100 mg/kg p.o, induced hyperlipidemic rat model *in vivo*	Dose = 200, 400, 800 mg/kg, p.o. *n* = 6	Cholesterol Dose = 100 mg/kg	↓cholesterol	[3]
		Triton-X 100, 480 mg/kg, i.p. induced hyperlipidemic rat model *in vivo*	Dose = 200, 400, 800 mg/kg, p.o. *n* = 6	Triton-X 100 Dose = 480 mg/kg	↓triglycerides ↓LDL-C ↓VLDL-C	

Antinociceptive	Hydroalcoholic extract (roots)	Acetic acid-induced writhing and hot plate-induced pain in mice *in vivo*	Dose = 250, 500 mg/kg b.w., i.p. *n* = 6	Diclofenac sodium Dose = 10 mg/kg	50% writhing inhibition ↑pain threshold	[46]
	Methanol extract (leaves)	Acetic acid writhing method and radiant heat tail flicking method *in vivo*	Dose = 200, 400 mg/kg, p.o. *n* = 5	Diclofenac sodium Dose = 50 mg/kg	↓number of writhes ↑reaction time at dose 400 mg/kg	[69]

Gastroprotective	Ethanol extract (leaves)	Indomethacin, 5 mg/kg b.w., p.o. Cold restraint stress-induced ulcers in rats *in vivo*	Dose = 100, 200, 400 mg/kg, p.o. *n* = 5	Indomethacin dose = 5 mg/kg	Development of a defensive layer; ↑free radical scavenging activity; ↓LPO	[70]

Reproductive activity	Hydroalcoholic extract (rhizomes)	Male rats *in vivo*	Dose = 5, 10, 20, 40 g/kg b.w., p.o. *n* = 5	Distilled water	↑serum testosterone	[64]

Abbreviations: ABTS: 2,2′-azino-bis (3-ethylbenzothiazoline-6-sulfonic acid; BHT: butylatedhydroxytoluene; DPPH: 1,1-diphenyl-2-pecrylhydrazyl; HO-1: heme-oxygenase-1; L-NAME: *N*-Nitro-L-arginine methyl ester; LDL-C: low-density lipoprotein cholesterol; MAPK: mitogen-activated protein kinase; NF-*κ*B: kappa B; Nrf2: nuclear factor erythroid 2-related factor 2; PI3K/Akt: phosphoinositide-3-kinase/protein kinase B; VLDL-C: very-low-density lipoprotein cholesterol.
